# Demographic history and gene flow during silkworm domestication

**DOI:** 10.1186/s12862-014-0185-0

**Published:** 2014-08-14

**Authors:** Shao-Yu Yang, Min-Jin Han, Li-Fang Kang, Zi-Wen Li, Yi-Hong Shen, Ze Zhang

**Affiliations:** 1 State Key Laboratory of Silkworm Genome Biology, The Key Sericultural Laboratory of Agricultural Ministry, Southwest University, Chongqing 400715, China; 2 Laboratory of Evolutionary and Functional Genomics, School of Life Sciences, Chongqing University, Chongqing 400044, China

**Keywords:** Coalescent simulation, Approximate Bayesian computation, Gene flow, Bombyx mori

## Abstract

**Background:**

Gene flow plays an important role in domestication history of domesticated species. However, little is known about the demographic history of domesticated silkworm involving gene flow with its wild relative.

**Results:**

In this study, four model-based evolutionary scenarios to describe the demographic history of *B. mori* were hypothesized. Using Approximate Bayesian Computation method and DNA sequence data from 29 nuclear loci, we found that the gene flow at bottleneck model is the most likely scenario for silkworm domestication. The starting time of silkworm domestication was estimated to be approximate 7,500 years ago; the time of domestication termination was 3,984 years ago. Using coalescent simulation analysis, we also found that bi-directional gene flow occurred during silkworm domestication.

**Conclusions:**

Estimates of silkworm domestication time are nearly consistent with the archeological evidence and our previous results. Importantly, we found that the bi-directional gene flow might occur during silkworm domestication. Our findings add a dimension to highlight the important role of gene flow in domestication of crops and animals.

## Background

The last decade has seen the advances in the field of demographic history involving gene flow of domesticated species [1],[2]. Initially, the studies of demographic history involving gene flow were mainly focused on crop plants [3]. The studies of domesticated animals, although they are crucial for human beings, are relatively lagged behind that of crop plants [4]. Until recently, some studies have investigated demography of domesticated animals, such as dog [5], chicken [6] and pig [7]. When considerable attention has been focused on these species, the same as domesticated species, few similar evolutionary studies have involved the domesticated silkworm, *Bombyx mori*.

The domesticated silkworm, *B. mori*, is an economically important insect, and widely distributed in many regions especially China, Korea, Japan and India [8]. In addition to its economic importance for farmers in these countries, the domesticated silkworm is a model of lepidopteran insects in basic research because of its short life cycle, and adaptation to laboratory culture [8]. The draft genome sequences of the domesticated silkworm were completed in 2004 [9],[10] and a new assembly of the genome sequence as well as the resequencing data were released in 2008 [11] and in 2009 [12]. These advances have also greatly promoted research on silkworm functional and evolutionary genomics.

With re-sequencing data of representative domesticated silkworm strains and wild silkworm samples from diverse geographic regions, the candidates of domestication genes were identified [12]. However, one puzzling observation in this study is that, although domesticated strains are clearly genetically differentiated from the wild ones, they still harbor ~83% of the variation observed in wild silkworm [12]. Later, using genetic evidence and coalescent simulation method, we showed that the domesticated silkworm lost 33-49% of nucleotide diversity relative to wild silkworm, which may attribute to a historical bottleneck during its domestication [13]. Furthermore, we investigated the phylogeny and evolutionary history of silkworm based on both mitochondrial loci and nuclear loci sequences [14]. However, some issues about inference of the demographic history involving gene flow of *B. mori* remain unresolved. Probably, the lack of sophisticated statistical methods limits the study of demographic history of silkworm. Fortunately, the latest Approximate Bayesian Computation (ABC) method is suitable for assuming different demographic models in Bayesican framework to infer evolutionary history of model species [15]–[17].

A growing number of studies have demonstrated that gene flow between wild and domesticated animal populations during domestication occurred frequently [18]. Typical evidence was found not only in pigs [19], sheep and goats [20], as well as Bactrian camel [21] but also in domesticated crop plants Genus *Zea*[1] and Pearl Millet [22]. However, the demographic history of silkworm involving gene flow has not been investigated. Because gene flow likely occurred in different phases of the whole demographic history of silkworm, in this study we first referred the evolutionary models of the crop species mentioned above and hypothesized different evolutionary models to describe the demographic history of *B. mori*. Then, we evaluated the most possible demographic scenario using the coalescent simulation and ABC method [23],[24] by combining the available nuclear DNA sequences and our newly sequenced data. Additionally, we also discussed the introgression pattern based on inferred evolutionary scenario with the goal of understanding the demographic history of silkworm.

## Results

### The characteristics of data used in this study

To avoid the effect of artificial selection and natural selection on the inference of the demographic history of silkworm as well as the detection of gene flow, we chose 17 loci that show neutral evolution in our previous studies [13],[25]. Furthermore, Tajima’s test [26] on each of 12 newly sequenced loci is not significant (Additional file 1), suggesting that these loci evolved in a neutral manner. Thus, all of 29 loci used in this study show a neutral evolution pattern.

### Demographic history of silkworm

**Figure 1:**
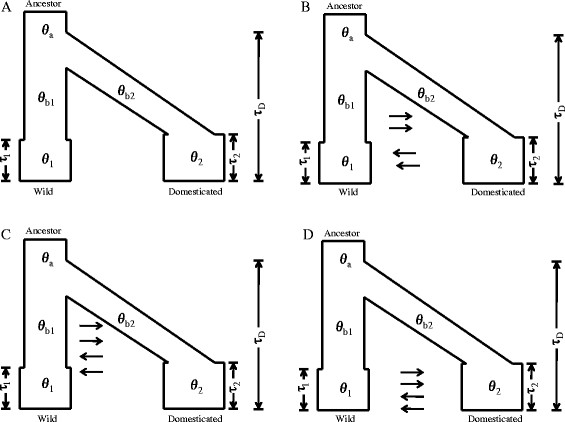
**Four demographic models. (A)** no gene flow. **(B)** continuous gene flow. **(C)** gene flow at bottleneck. **(D)** gene flow after bottleneck.

To investigate the occurrence of gene flow between the domesticated and wild silkworms, the demographic history of silkworm domestication was estimated by using coalescent simulation and ABC method. Four models (‘no gene flow’ model, ‘continuous gene flow’ model, ‘gene flow at bottleneck’ model and ‘gene flow after bottleneck’ model) were assumed (Figure 1). Eight demographic parameters (θ_1_, θ_2_/θ_1_, θ_a_/θ_1_, θ_b2_/θ_1_, θ_b1_/θ_1_, τ_D_, τ_1_ and τ_2_) were estimated. The posterior density curve of each parameter for each model was also calculated (Additional files 2, 3, 4 and 5). These parameters can be converted into effective population size (*Ne*) and time in years using the formulation of θ = 4*Neμ* and in units of 4 *N*_1_ generations, assuming the mutation rate *μ* = 1.56 × 10^−8^/bp/generation [10]. The values of these parameters are calculated (Additional file 6). We found that four models have very different parameter values. For example, *N*_b2_ is equal to ~10,793 individuals (95% confidence interval (CI): 2,075 – 95,560) in the ‘no gene flow’ model, ~9,310 individuals (95% CI: 2,100 – 65,250) in the ‘continuous gene flow’ model, ~7,669 individuals (95% CI: 1,390 – 63,205) in the ‘gene flow at bottleneck’ model, and ~8,279 individuals (95% CI: 1,785 – 64,820) in the ‘gene flow after bottleneck’ model; *T*_D_ is equal to ~6,334 years (95% CI: 3,000 – 55,120) in the ‘no gene flow’ model, ~7,424 years (95% CI: 3,080 – 57,800) in the ‘continuous gene flow’ model, ~7,460 years (95% CI: 2,640 – 56,680) in the ‘gene flow at bottleneck’ model, and ~5,906 years (95% CI: 3,360 – 63,560) in the ‘gene flow after bottleneck’ model.

To determine which model is the most likely scenario for silkworm domestication, the value of Bayes factor (*K*) was calculated. The results showed that the maximum value of *K* was produced by ‘gene flow at bottleneck’ model (Table 1). According to this model, the scenario for silkworm domestication was as follows. The effective population sizes of the domesticated silkworm population at the modern and bottleneck stages are 73,196 (95% confidence interval (CI): 10,270 – 549,065) and 7,669 (95% CI: 1,390 – 63,205), respectively. Additionally, we also estimated the divergence time: the starting time of the domestication is ~7,460 years ago (95% CI: 2,640 – 56,680); the time of bottleneck ended in the domesticated silkworm is ~3,984 years ago (95% CI: 1,980 – 42,900) (Table 2). This model showed that gene flow occurred at the bottleneck stage during silkworm domestication. However, the direction of gene flow between the domesticated silkworm and wild silkworm is still unknown.

**Table 1:** Estimates of Bayes factor for hypothesized models

**Statistic of demographic inference**	**Model**	**K**
π_silent-D_	A: No gene flow	1
	B: Continuous gene flow	80.5
	C: Gene flow at bottleneck	293.63
	D: Gene flow after bottleneck	8.8
S_silent-D_	A: No gene flow	1
	B: Continuous gene flow	109
	C: Gene flow at bottleneck	185.26
	D: Gene flow after bottleneck	11.3

K: Bayes factor; π_silent-D_: π_silent_ values for *B. mori*; S_silent-D_: S_silent_ values for *B. mori.*

**Table 2:** Silkworm domestication history parameters of preferred model

**Parameters**	**Max**	**Lower 95%**	**Upper 95%**
θ_a_	0.98953	0.86365	1.16068
*N*_a_	494,765	431,825	580,340
θ_2_	0.146392	0.02054	1.09813
*N*_2_	73,196	10,270	549,065
θ_b1_	0.94133	0.11927	1.31742
*N*_b1_	470,665	59,635	658,710
θ_b2_	0.015339	0.00278	0.12641
*N*_b2_	7,669	1,390	63,205
τ_D_	0.00373	0.00132	0.02834
*T*_D_	7,460	2,640	56,680
τ_1_	0.0025	0.00107	0.02438
*T*_1_	5,000	2,140	48,760
τ_2_	0.001992	0.00099	0.02145
*T*_2_	3,984	1,980	42,900

Parameters of the preferred model were estimated. θ and τ are converted to *N* and *T* (years) respectively. Conversion procedure is based on the equation θ = 4*N*_*1*_μ, neutral mutation rate μ = 1.56 × 10^−8^ per site per generation was used.

### Detection of gene flow direction

**Figure 2:**
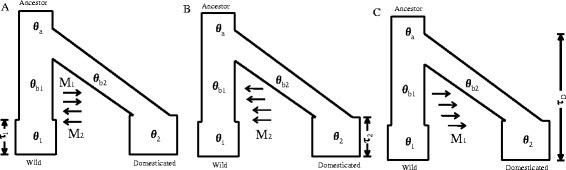
**Modeling the gene flow directions of the domesticated (*B. mori*) and wild (*B. mandarina*) silkworms based on gene flow at bottleneck model. (A)** Bidirectional gene flow between the domesticated and wild populations. **(B)** Gene flow from *B. mori* into *B. mandarina*. **(C)** Gene flow from *B. mandarina* into *B. mori*.

To determine the direction of gene flow between the two populations, three models were assumed (Figure 2). Based on these models, simulations were performed by using the software of MIGRATE 3.2.16. The results showed that the model probabilities of Model A, Model B and Model C are 0.9999, 0.0001 and 0.0000, respectively (Table 3). This indicates that Model A with the bidirectional gene flow during bottleneck is the most likely scenario. Accordingly, we estimated the migration rates. The migration rate from wild to domesticated silkworms is 107.1 (95% CI: 58.3 ~ 153.8) (number of migrants per generation) while the migration rate from domesticated to wild silkworms is 318.6 (95% CI: 237.2 ~ 397.4). To further confirm bidirectional gene flow occurred during silkworm domestication, the IMa program was used to perform simulation analysis [27],[28]. Likelihood-ratio test on bidirectional migration between ancestral sub-populations is significant (*P* < 0.001). The maximum likelihood estimates of the bidirectional migration rate from wild to domesticated silkworm population (m_1_) and from domesticated to wild silkworm population (m_2_) are 0.25 (95% CI: 0.0235 ~ 0.9735) and 0.91 (95% CI: 0.0352 ~ 1.4603) (per mutation), respectively (Additional file 7). This further suggests that the bidirectional migration might occur during silkworm domestication. Furthermore, the results of both approaches suggested that the migration rate from domesticated to wild silkworms is larger than that from wild to domesticated silkworms.

**Table 3:** Log marginal likelihood values, LBF and model probability of migration models

**Model**	**Thermodynamic score**	**LBF**	**Relative model probability**
D ← →W	−50024.87	0.00	0.9999
D → W	−50032.70	−7.83	0.0001
W → D	−50052.43	−19.82	0.0000

D: *B. mori*; W: *B. mandarina*; LBF: log Bayes factor.

## Discussion

Since the genome sequence of the domesticated silkworm was completed, many studies have used the genome-wide nuclear data and the classic sequencing data of individual nuclear loci to infer the demography and domestication of silkworm [12]–[14],[25],[29]. Although these studies provide a lot of useful information for understanding the origin and domestication of the domesticated silkworm, they appear to ignore detection of gene flow between the domesticated and wild populations.

Previous studies based on the nuclear genome data suggested that the domesticated silkworm lost only approximately 17% of nucleotide diversity measured by θ relative to wild silkworm [12]. However, our previous studies revealed that the domesticated silkworm lost 33-49% of nucleotide diversity measured by θ relative to wild silkworm and experienced a severe bottleneck during domestication [13],[14],[25]. As indicated previously, the main reason leading to this big difference is that studies based on Solexa sequencing data might have underestimated the level of nucleotide diversity in wild silkworm [13]. The nucleotide diversity in the domesticated silkworm was found to be at a very similar level measured as θ between the classic sequencing data and Solexa resequencing data, however, the nucleotide diversity in wild silkworm was greatly underestimated when the Solexa resequencing data were used [13]. The possible reason is that the genome sequence of wild silkworm, *B. mandarina*, has not been available to date, thus, the genome sequence of the domesticated silkworm was used as a reference for calling SNPs in both the domesticated and wild silkworms. Because resequencing reads are usually short (~45 bp), some mapping algorithms cannot map sequence reads with more than one or two differences from a reference genome sequence [30]. Thus, we decided to reconsider the demographic history of the domesticated silkworm using a combined data set of nuclear loci by classic sequencing rather than the whole genome resequencing data by Solexa. It will be good time to better infer the demographic history of the domesticated silkworm using the whole genome resequencing data only when the genome sequence of wild silkworm, *B. mandarina*, is available in future.

Since previous studies have ignored the detection of gene flow between the domesticated and wild silkworms [12]–[14],[25], this study intended to detect possible introgression pattern of the domesticated and wild populations. Currently, very few studies had investigated the evolutionary pattern of the domesticated silkworm, *B. mori*. Therefore, quite limited available model information could be used when we started to investigate the demographic history of the domesticated silkworm. Using previous studies on historical divergence and gene flow of genus *Zea* and Pearl Millet as references [1],[22], therefore, we hypothesized four evolutionary scenarios by adding gene flow to reconsider the silkworm’s domestication. We found that the ‘gene flow at bottleneck’ model is the most likely scenario. According to this model, the starting time of the silkworm domestication is ~7,460 years ago (95% CI: 2,640 – 56,680); the time of bottleneck ended in the domesticated silkworm is ~3,984 years ago (95% CI: 1,980 – 42,900). These results are consistent with our previous results based on the phylogenetic analyses in which the domestication of silkworm was estimated to occur about 4,100 years ago [14]. In addition, the estimate of the silkworm domestication time is also well consistent with the domestication time estimates of rice (~7,500 years) as well as maize (~7,500 years) [31],[32]. These results are nearly consistent with the evidence from fossils [33]. In addition, the domestication time of most livestock can be traced back to 8,000-10,000 years ago [34]. Our divergence timing estimation is also within the timeframe. Therefore, our results are reasonable.

Coalescent simulation indicated that the gene flow might occur during the period of bottleneck. Three possible reasons may explain this. First, the overlapping geographic regions between the domesticated and wild silkworm populations might facilitate the gene flow [35]. Wild silkworm is not only widely distributed in the regions of Jiangsu and Zhejiang provinces, but also can be found in Anhui and Shandong provinces in China. Interestingly, these areas are also the main regions of sericulture [36]. Second, the lack of the absolute reproductive isolation is likely to be another important factor that leads to gene flow between the populations. In fact, the domesticated and wild silkworms have not formed the completely reproductive isolation up to now. They are able to interbreed with each other. This may provide some convenience for gene exchanging between these two species. Third, the environmental factors may also provide opportunities for gene flow between the populations during silkworm domestication. The initial rearing conditions of the domesticated silkworm may be very simple and open compared with present-day’s rearing environments [36]. This may also increase the opportunities of contact and hybridization between the domesticated and wild silkworms. Besides these factors, economic expansion and urbanization might also affect gene flow. Rapid economic expansion and intensive urbanization may be common causes of the habitat fragmentation and may eventually reduce the gene flow on wildlife populations between different fragmented landscapes [37],[38]. Thus economic expansion and urbanization could be the reasons of why gene flow occurred at the period of bottleneck rather than at the recent.

To determine the direction of gene flow during bottleneck, simulations were performed by using the software of MIGRATE 3.2.16. The results indicated that bidirectional gene flow during the bottleneck period between the populations is the most likely scenario (Table 3). In addition, the IM simulation results also supported the bidirectional gene migration between the domesticated and wild silkworm populations rather than the unidirectional gene flow model (Additional file 7). The gene flow direction from the domesticated silkworm to wild silkworm might be caused by the situation that the domesticated silkworms were discarded together with their excrement into natural environment [36]. The gene flow from wild silkworm to the domesticated silkworm might be because of that wild silkworm was introduced by breeders, as an important genetic source, to hybrid with the domesticated silkworm to produce desirable strains [8],[39].

In this study, we revealed that there was gene flow between the domesticated and wild silkworms. Our finding is consistent with the observations in maize [1], pig [19],[40], chicken [41], cattle [42],[43], cats [44], horses [45], and honeybee [46]. It appears that gene flow is ubiquitous between domesticated species and their wild relatives. Our results may add a dimension to highlight the important role of gene flow in domestication of crops and animals. Furthermore, as more and more population genomics data become available and new statistical methods are developed, we believe that long-standing questions about the role of gene flow in species formation will be resolved soon [47].

## Conclusions

In this study, we hypothesized different models to describe the demographic history and the gene flow between *B. mori* and its wild relative, *B. mandarina*. The starting time of silkworm domestication was estimated to be approximate 7,500 years ago; the time of domestication termination was 3,984 years ago. Bi-directional gene flow might occur during silkworm domestication. Our findings add a dimension to highlight the important role of gene flow in domestication of crops and animals.

## Methods

### Data sources

In previous studies, we sequenced 17 loci in the domesticated silkworm (6 to 17 strains) and wild silkworm (7 to 15 samples) [13],[25]. To expand our data set, we sequenced another 12 nuclear loci in this study, in total the current data set includes 29 loci DNA sequences (Additional file 8). The domesticated and wild silkworms used in this study are almost the same as those used in previous study [13],[25]. All new sequences have been submitted to GenBank (Accession nos. KC137982-KC138225, KF703556-KF703614). The domesticated silkworm strains were randomly obtained from the Institute of Sericulture and Systems Biology at Southwest University, China, and represented the four main geographic strains (Chinese, Japanese, European and Tropical). Wild silkworm samples were collected from various geographical regions in China. No specific permissions were required for sampling wild silkworm. Sequences were aligned by BioEdit 7.0.4.1 [48]. For each locus, π_silent_ (π values for synonymous and noncoding sites) and θ_silent_ (θ values for synonymous and non-coding sites) were calculated using DNAsp 5.00 [49].

### Demographic history analysis

The coalescent simulation was performed using ms software [50] and the four models (‘no gene flow’ model, ‘continuous gene flow’ model, ‘gene flow at bottleneck’ model and ‘gene flow after bottleneck’ model) were introduced (Figure 1). No gene flow model assumes no gene flow between the domesticated silkworm and wild silkworm populations. Continuous gene flow model assumes continuous gene flow between the populations. In the ‘gene flow at bottleneck’ model, gene flow occurred only in the bottleneck period. In the ‘gene flow after bottleneck’ model, gene flow occurred after the end of bottleneck period. For all four models, eight demographic parameters (θ_1_, θ_2_/θ_1_, θ_a_/θ_1_, θ_b2_/θ_1_, θ_b1_/θ_1_, τ_D_, τ_1_ and τ_2_) were calculated (the ancestral population evolved with a population mutation rate θ_a_ and the ancestral population spitted into two bottlenecked populations of size θ_b1_ and θ_b2_ at time τ_D_. The wild silkworm population θ_1_ was assumed as reference population. The daughter populations remained small until they recovered from their bottlenecks to modern sizes of θ_1_ and θ_2_ at times τ_1_ and τ_2_ in the past). In our simulations, prior values and distribution of the parameters were listed in Additional file 9. The prior values were referenced the previous studies in model organisms [1],[17]. For each model, 2,000,000 simulations were performed using the ms software [48]. To summarize the dataset, we made use of two statistics (the number of segregating sites at silent sites (*S*_silent_) and the average number of pairwise differences at silent sites (*π*_silent_) for each population) for demographic inference. The posterior probability distributions of eight parameters were calculated using rejection-regression procedure [15] and acceptance values of 0.001 were used for all analyses. Regression analysis was performed using the program ABCreg [51].

To determine which of the above four assumed models is the most likely scenario for silkworm domestication, Bayes factor was calculated using the following method [1]. Briefly, the values of the domesticated silkworm *S* and π were simulated using the program ms [50] with the parameter values drawn from the posterior distributions of the demographic models inferred by use of the 29 loci sequences. For each model, the acceptable simulation of each model was defined based on the Euclidean distance δ = 0.01 (| (simulated values - observed values)/observed values | < δ). Furthermore, the acceptance rate of each model was calculated. Finally, we assumed the isolation model as a reference model. The relative acceptance rates (the Bayes factor (*K*)) of each model were also calculated.

### Detecting the direction of gene flow between populations

To determine the directional gene flow during bottleneck, MIGRATE 3.2.16 was used to perform analysis [52]. Three models based on inferred model were introduced. These models are shown in Figure 2. (1) Model A assumes two directional introgressions (one from the domesticated silkworm into wild silkworm population and the other from wild silkworm into the domesticated silkworm population); (2) Model B assumes only unidirectional introgression (from domesticated silkworm into wild silkworm population); (3) Model C also assumes unidirectional introgression (from wild silkworm into the domesticated silkworm population). All parameters of the models are described in Additional file 10. For each model, four parallel static chains were performed with temperatures of 1.0, 1.5, 3.0 and 10^4^, with a number of 10,000 recorded steps and with 100 replicate runs. The log marginal likelihood values for the Bezier approximated score and the Harmonic mean were calculated by MIGRATE software. Furthermore, the probability of each model and log Bayes Factors (LBF) were calculated using the formulations as follows: (1) $\mathrm{Prob}\;\left({\mathrm{model}}_{i}\right)=\mathrm{mL}{\mathrm{model}}_{i}/\sum_{j}^{n}\;\mathrm{mL}{\mathrm{model}}_{j}$; (2) LBF = ln (mL (model1)) − ln (mL (model2)). In addition, to further confirm the direction of gene flow during bottleneck, we also used the isolation with migration (IM) model as implemented in IMa software [27] to detect the possible gene flow directions between these two closely related populations. IM model also assumes the ancestral population splits into two descendant sub-populations whose sub-population can migrate with each other. IMa software is based on Markov Chain Monte Carlo (MCMC) to simulate gene genealogies. We performed Markov Chain Monte Carlo (MCMC) simulation with a million burn-in period followed by a million runs to generate genealogies. Our runs were conducted using the identical setting but with three different random number seeds. Furthermore, the estimates of joint-posterior densities and likelihood-ratio tests (LRT) were also performed to select the most likely model. Finally, based on IM model, the migration parameters from the wild to domesticated silkworm population (m_1_) and the migration parameter from the domesticated silkworm to wild silkworm population (m_2_) were also calculated.

## Competing interests

The authors declare that they have no competing interests.

## Authors’ contributions

YHS and ZZ designed the study. SYY and MJH performed the analyses and drafted the manuscript. LFK and ZWL performed the experiments. YHS and ZZ supervised the study and revised the manuscript. All authors read and approved the final manuscript.

## Additional files

## Supplementary Material

Additional file 1: Table S1. Summary statistics of nucleotide diversity and neutrality test results of 12 loci sequenced in this study. Click here for file

Additional file 2: Figure S1. Posterior distributions of demographic parameters in no gene flow model. Click here for file

Additional file 3: Figure S2. Posterior distributions of demographic parameters in continuous gene flow model. Click here for file

Additional file 4: Figure S3. Posterior distributions of demographic parameters in gene flow at bottleneck model. Click here for file

Additional file 5: Figure S4. Posterior distributions of demographic parameters in gene flow after bottleneck model. Click here for file

Additional file 6: Table S2. Estimation values of parameters for hypothesized four demographic models. Click here for file

Additional file 7: Figure S5. Maximum likelihood estimates of migration parameters using IMa analysis. Click here for file

Additional file 8: Table S3. Summary of all loci information of *B. mori* and *B. mandarin.* Click here for file

Additional file 9: Table S4. Priors used for Approximate Bayesian Computation. Click here for file

Additional file 10: Table S5. Parameters of all hypothesized models. Click here for file
