# Supplementary material for: Demographic history and gene flow during silkworm domestication
Source: BMC Evol Biol. 2014 Aug 14;14:185. doi: 10.1186/s12862-014-0185-0 (PMC4236568; doi:10.1186/s12862-014-0185-0)
Supplement: Additional file 1: Table S1. — Summary statistics of nucleotide diversity and neutrality test results of 12 loci sequenced in this study. [file s12862-014-0185-0-S1.doc]

**Table S1:** Summary statistics of nucleotide diversity and neutrality test results of 12 loci sequenced in this study.

| **gene** | **speies** | **n** | **Length** | **S** | **π_total_** | **θ_w_** | **R_m_** | **R** | **Tajima's D** |
| --- | --- | --- | --- | --- | --- | --- | --- | --- | --- |
| *P450* | Domesticated | 9 | 402 | 33 | 0.03538 | 0.03020 | 3 | 0.0177 | 1.23376 |
|  | Wild | 7 | 402 | 48 | 0.04525 | 0.04874 | 3 | 0.1793 | -0.31447 |
| *CBF* | Domesticated | 7 | 1034 | 47 | 0.01483 | 0.01855 | 0 | 0.0040 | -1.15187 |
|  | Wild | 8 | 1034 | 73 | 0.02311 | 0.02723 | 5 | 0.1801 | -0.70385 |
| *TPK* | Domesticated | 8 | 735 | 51 | 0.02284 | 0.02676 | 4 | 0.0185 | -0.66748 |
|  | Wild | 7 | 735 | 38 | 0.02086 | 0.02110 | 3 | 0.0594 | 0.07643 |
| *TFIID* | Domesticated | 6 | 953 | 14 | 0.00644 | 0.00643 | 0 | 0.0203 | 0.00195 |
|  | Wild | 7 | 953 | 39 | 0.01539 | 0.01670 | 1 | 0.0232 | -0.45205 |
| *Bmo -mir-285* | Domesticated | 17 | 432 | 19 | 0.01917 | 0.01354 | 0 | 0 | 1.63758 |
|  | Wild | 11 | 432 | 44 | 0.03383 | 0.03602 | 7 | 0.3068 | -0.38444 |
| *Bmo-mir-2794* | Domesticated | 17 | 463 | 13 | 0.0128 | 0.01039 | 0 | 0.0019 | 0.8771 |
|  | Wild | 11 | 463 | 31 | 0.01689 | 0.02594 | 0 | 0.0653 | -1.71629 |
| *Bmo-mir-2795* | Domesticated | 17 | 344 | 15 | 0.00784 | 0.01324 | 2 | 0 | -1.72658 |
|  | Wild | 10 | 344 | 20 | 0.02006 | 0.02216 | 4 | 0.0409 | -0.44531 |
| *Bmo-mir-2822-1* | Domesticated | 17 | 254 | 11 | 0.00996 | 0.01281 | 0 | 0 | -0.82602 |
|  | Wild | 11 | 254 | 17 | 0.01926 | 0.02294 | 0 | 0.3024 | -0.71864 |
| *Bmo-mir-2823* | Domesticated | 17 | 343 | 38 | 0.03956 | 0.03287 | 6 | 0.0211 | 0.71372 |
|  | Wild | 11 | 343 | 38 | 0.0352 | 0.03782 | 6 | 0.0223 | -0.32507 |
| *Bmo-mir-2827* | Domesticated | 16 | 459 | 3 | 0.00169 | 0.00197 | 0 | 0.0031 | -0.41395 |
|  | Wild | 9 | 459 | 5 | 0.00351 | 0.00401 | 0 | Non | -0.52629 |
| *Bmo-mir-2831-2* | Domesticated | 16 | 380 | 18 | 0.01638 | 0.01435 | 0 | 0 | 0.56374 |
|  | Wild | 10 | 380 | 27 | 0.02556 | 0.02518 | 1 | 0.0591 | -0.10094 |
| *Bmo-mir-2837* | Domesticated | 17 | 307 | 16 | 0.02643 | 0.01746 | 1 | 0.0048 | 1.35321 |
|  | Wild | 9 | 307 | 24 | 0.03321 | 0.03258 | 2 | 0.0284 | 0.09533 |

n: sample size; length: the number of sites aligned sequences; S: the number of segregating sites; π_total_: the mean number of nucleotide differences per site; θ_w_: watterson’s estimator of 4Neμ; θ_silent_: θ values for silent sites (synonymous and noncoding sites); π_silent_: π values for or silent sites (synonymous and noncoding sites); R_m_: estimates of minimum number of recombination events (Hudson and Kaplan 1985); R: the estimate of population recombination parameter per site (Hudson 1987).
