# Supplementary figures and images for: Demographic history and gene flow during silkworm domestication
Source: BMC Evol Biol. 2014 Aug 14;14:185. doi: 10.1186/s12862-014-0185-0 (PMC4236568; doi:10.1186/s12862-014-0185-0)

Figure S1

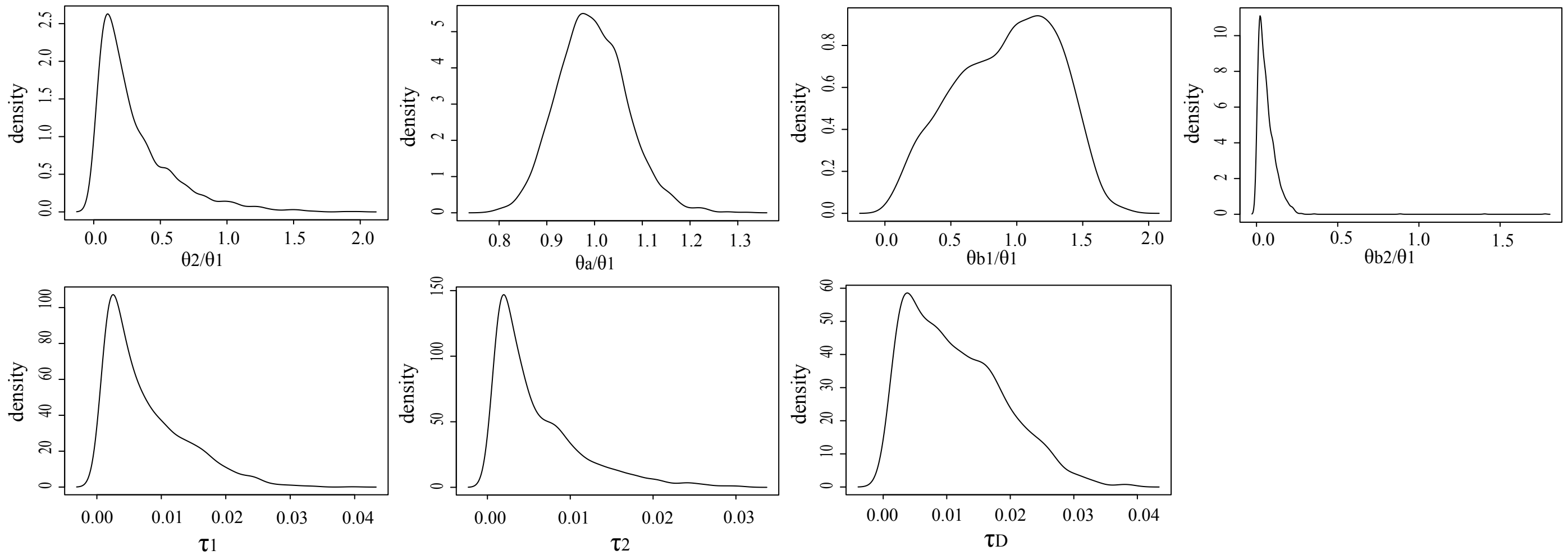

Supplement: Additional file 2: Figure S1. — Posterior distributions of demographic parameters in no gene flow model. [file s12862-014-0185-0-S2.pdf]

Figure S2

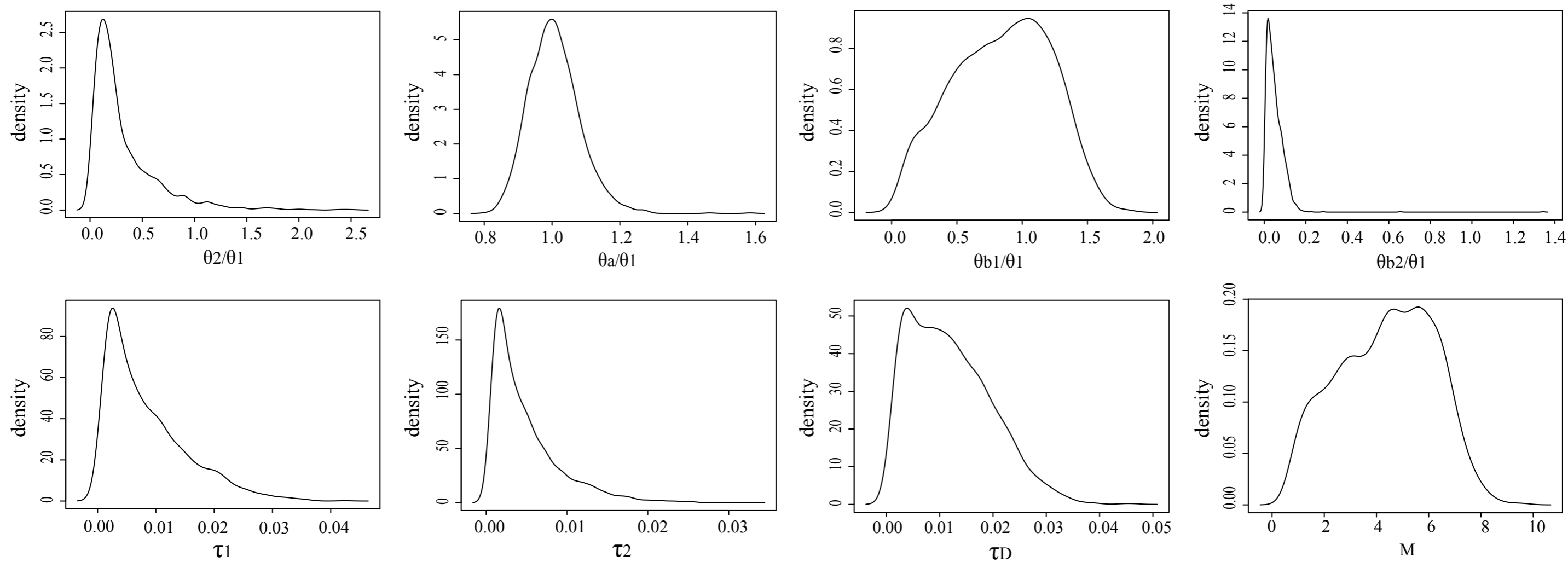

Supplement: Additional file 3: Figure S2. — Posterior distributions of demographic parameters in continuous gene flow model. [file s12862-014-0185-0-S3.pdf]

Figure S3

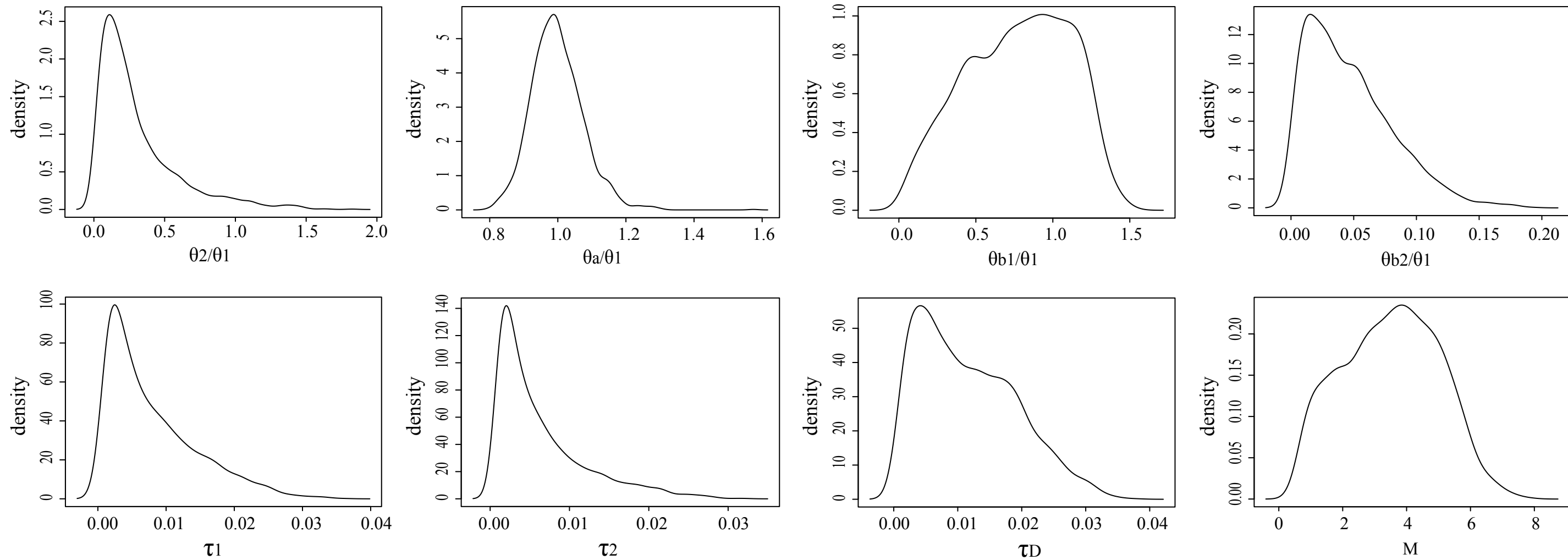

Supplement: Additional file 4: Figure S3. — Posterior distributions of demographic parameters in gene flow at bottleneck model. [file s12862-014-0185-0-S4.pdf]

Figure S4

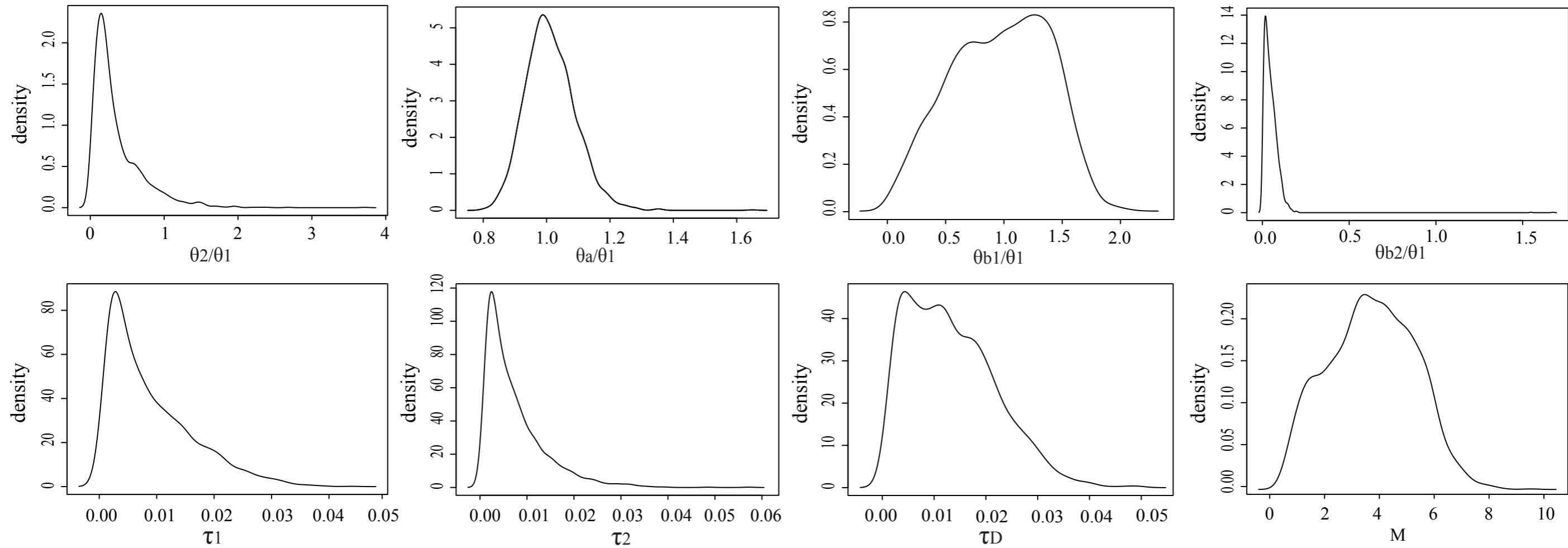

Supplement: Additional file 5: Figure S4. — Posterior distributions of demographic parameters in gene flow after bottleneck model. [file s12862-014-0185-0-S5.pdf]

Figure S 5

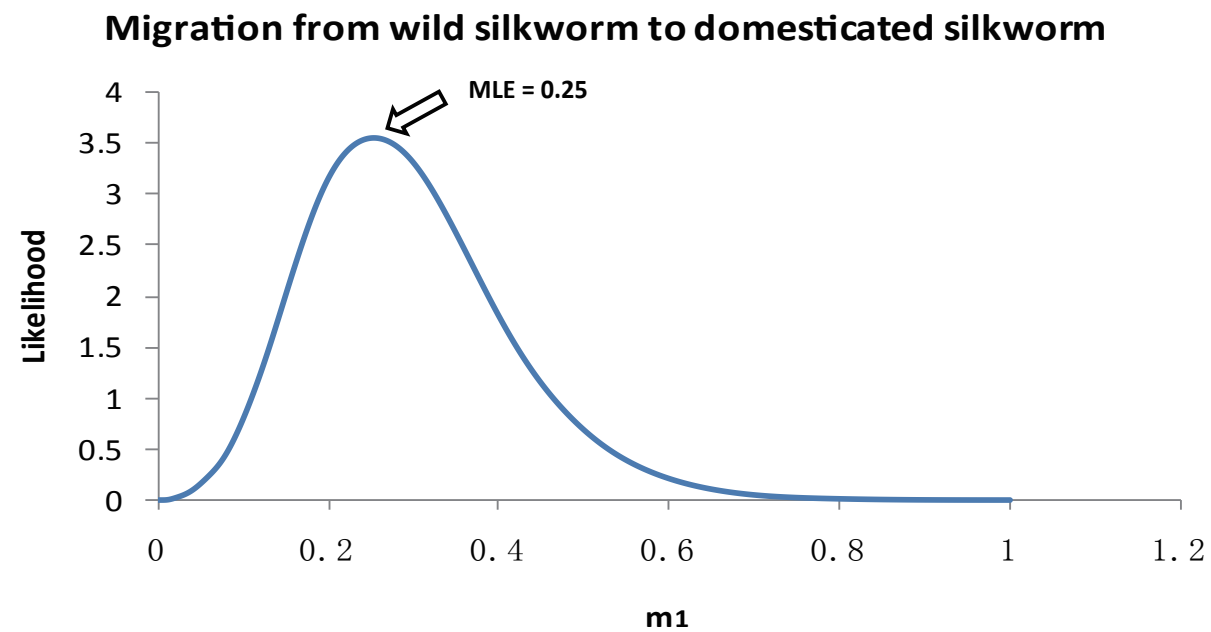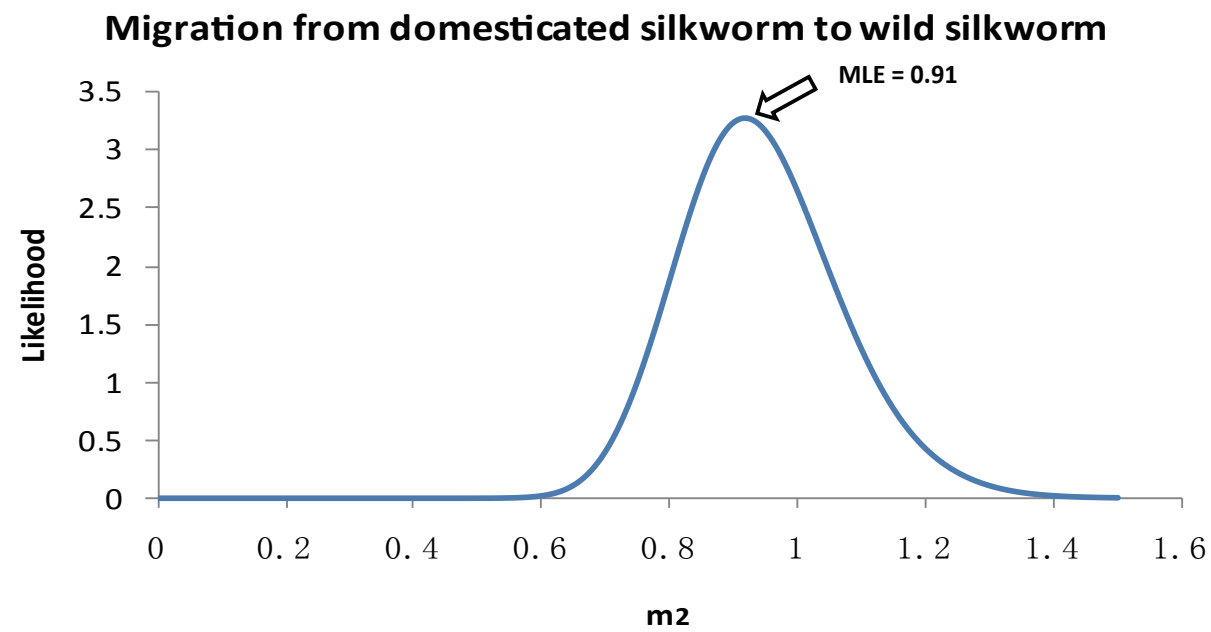

Supplement: Additional file 7: Figure S5. — Maximum likelihood estimates of migration parameters using IMa analysis. [file s12862-014-0185-0-S7.pdf]
