# Supplementary material for: Demographic history and gene flow during silkworm domestication
Source: BMC Evol Biol. 2014 Aug 14;14:185. doi: 10.1186/s12862-014-0185-0 (PMC4236568; doi:10.1186/s12862-014-0185-0)
Supplement: Additional file 6: Table S2. — Estimation values of parameters for hypothesized four demographic models. [file s12862-014-0185-0-S6.doc]

Table S2. Estimation values of parameters for hypothesized four demographic models

|  | model | | | |
| --- | --- | --- | --- | --- |
| Parameters | No gene flow | Continuous gene flow | Gene flow at bottleneck | Gene flow after bottleneck |
| θ_a_ | 0.9692 (0.89354-1.15) | 0.98728 (0.8775-1.17) | 0.98953 (0.86365-1.16068) | 0.988734 (0.87485-1.1809) |
| *N*_a_ | 484,600 (446,770-57,515) | 493,640 (438,750-585,350) | 494,765 (431,825-580,340) | 494,367 (437,426-590,450) |
| θ_2_ | 0.08229 (0.02393-1.0794) | 0.121178 (0.2565-1.2213) | 0.146392 (0.02054-1.09813) | 0.173553 (0.02666-1.29) |
| *N*_2_ | 41,145 (11,960-539,716) | 60,589 (12,825-610,650) | 73,196 (10,270-549,065) | 86,776 (13,330-645,000) |
| θ_b1_ | 1.01464 (0.19483-1.55247) | 1.044 (0.1369-1.4831) | 0.94133 (0.11927-1.31742) | 1.1663 (0.17896-1.67638) |
| *N* _b1_ | 507,320 (9,745-15-776,235) | 522000 (68,450-741,550) | 470,665 (59,635-658,710) | 583,150 (89,480-838,190) |
| θ_b2_ | 0.021587 (0.00415-0.19112) | 0.01862 (0.0042-0.1305) | 0.015339 (0.00278-0.12641) | 0.016559 (0.00357-0.12964) |
| *N* _b2_ | 10,793 (2,075-95,560) | 9,310 (2,100-65,250) | 7,669 (1,390-63,205) | 8,279 (1,785-64,820) |
| τ_D_ | 0.003167 (0.0015-0.02756) | 0.003712 (0.00154-0.0289) | 0.00373 (0.00132-0.02834) | 0.002953 (0.00168-0.03178) |
| *T*_D_ | 6,334 (3,000-55,120) | 7,424 (3,080-57,800) | 7,460 (2,640-56,680) | 5,906 (3,360-63,560) |
| τ_1_ | 0.001702 (0.001-0.02333) | 0.001333 (0.01126-0.02574) | 0.0025 (0.00107-0.02438) | 0.001672 (0.001127-0.02742) |
| *T*_1_ | 3,404 (2,000-46,660) | 2,667 (2,252-51,480) | 5,000 (2,140-48,760) | 3,344 (2,540-54,840) |
| τ_2_ | 0.0014 (0.000965-0.02058) | 0.00088 (0.00077-0.01647) | 0.001992 (0.00099-0.02145) | 0.001351 (0.00116-0.0245) |
| *T*_2_ | 2,800 (1,930-41,160) | 1,760 (1,540-32,940) | 3,984 (1,980-42,900) | 2,702 (2,230-49,000) |

Parameters of hypothesized models are shown above. θ and τ are converted to *Ns* and *Ts* (years) respectively. Conversion procedure is based on the equation θ = 4*N*_1_μ, and *T* = 4 *N*_1_τ, neutral mutation rate μ=1.56×10^-8^ per site per generation was used. The reference effective population size *N_1_* = 5×10^5^ was used.
