# Supplementary material for: Demographic history and gene flow during silkworm domestication
Source: BMC Evol Biol. 2014 Aug 14;14:185. doi: 10.1186/s12862-014-0185-0 (PMC4236568; doi:10.1186/s12862-014-0185-0)
Supplement: Additional file 8: Table S3. — Summary of all loci information of B. mori and B. mandarin. [file s12862-014-0185-0-S8.doc]

Table S3. Summary of all loci diversity of *B. mori* and *B. mandarina*

| **Gene** | **Populations** | **n** | **π_silent_** | **θ_silent_** | **Accession number** | **Reference** |
| --- | --- | --- | --- | --- | --- | --- |
| *Wnt-1* | Domesticated | 16 | 0.01209 | 0.0135 | GQ420700-GQ420730 | Guo et al., 2011 |
|  | Wild | 15 | 0.01464 | 0.02396 |  |  |
| *ER* | Domesticated | 16 | 0.0035 | 0.00506 | GQ420731-GQ420761 | Guo et al., 2011 |
|  | Wild | 15 | 0.00622 | 0.0109 |  |  |
| *GST* | Domesticated | 16 | 0.01729 | 0.01955 | GQ420762-GQ420792 | Guo et al., 2011 |
|  | Wild | 15 | 0.02379 | 0.03702 |  |  |
| *α-Amy* | Domesticated | 15 | 0.01867 | 0.02208 | GQ420821-GQ420850 | Guo et al., 2011 |
|  | Wild | 15 | 0.029 | 0.0348 |  |  |
| *Adh* | Domesticated | 14 | 0.00809 | 0.00956 | GQ420793-GQ420820 | Guo et al., 2011 |
|  | Wild | 14 | 0.01696 | 0.02562 |  |  |
| *PBAN* | Domesticated | 16 | 0.02676 | 0.02154 | GQ423313-GQ423341 | Guo et al., 2011 |
|  | Wild | 13 | 0.03639 | 0.03886 |  |  |
| *RpSA* | Domesticated | 14 | 0.03221 | 0.02404 | HM132882-HM132909 | Guo et al., 2011 |
|  | Wild | 14 | 0.0349 | 0.03386 |  |  |
| *AchE* | Domesticated | 11 | 0.02969 | 0.01909 | HM132910-HM132933 | Guo et al., 2011 |
|  | Wild | 13 | 0.03886 | 0.04219 |  |  |
| *CecE* | Domesticated | 15 | 0.03457 | 0.03659 | HQ203989-HQ204015 | Guo et al., 2011 |
|  | Wild | 12 | 0.0442 | 0.05778 |  |  |
| *MorB3* | Domesticated | 13 | 0.02251 | 0.01541 | HQ204016-HQ204039 | Guo et al., 2011 |
|  | Wild | 11 | 0.01697 | 0.01283 |  |  |
| *Black* | Domesticated | 16 | 0.00058 | 0.00135 | HM545477-HM545506 | Yu et al.,2011 |
|  | Wild | 14 | 0.02570 | 0.03129 |  |  |
| *Ddc* | Domesticated | 15 | 0.01407 | 0.01110 | HM545597-HM545626 | Yu et al.,2011 |
|  | Wild | 15 | 0.02686 | 0.03493 |  |  |
| *Ebony* | Domesticated | 15 | 0.00925 | 0.01070 | HM545657-HM545686 | Yu et al.,2011 |
|  | Wild | 15 | 0.03090 | 0.03113 |  |  |
| *Yellow-f* | Domesticated | 15 | 0.04451 | 0.03575 | HM545567-HM545596 | Yu et al.,2011 |
|  | Wild | 15 | 0.05195 | 0.04861 |  |  |
| *Dat* | Domesticated | 15 | 0.02146 | 0.01978 | HM545627-HM545656 | Yu et al.,2011 |
|  | Wild | 15 | 0.02584 | 0.03298 |  |  |
| *Yellow* | Domesticated | 16 | 0.02271 | 0.02685 | HM545537-HM545566 | Yu et al.,2011 |
|  | Wild | 14 | 0.03071 | 0.03564 |  |  |
| *tan* | Domesticated | 15 | 0.03319 | 0.03294 | HM545687-HM545716 | Yu et al.,2011 |
|  | Wild | 15 | 0.04104 | 0.05365 |  |  |
| *P450* | Domesticated | 9 | 0.05216 | 0.04338 | KF703586-KF703601 | This study |
|  | Wild | 7 | 0.06215 | 0.0653 |  |  |
| *CBF* | Domesticated | 7 | 0.01805 | 0.02256 | KF703556-KF703570 | This study |
|  | Wild | 8 | 0.02915 | 0.03452 |  |  |
| *TPK* | Domesticated | 8 | 0.02551 | 0.02977 | KF703571-KF703585 | This study |
|  | Wild | 7 | 0.02377 | 0.02346 |  |  |
| *TFIID* | Domesticated | 6 | 0.00644 | 0.00643 | KF703602-KF703614 | This study |
|  | Wild | 7 | 0.01539 | 0.0167 |  |  |
| *Bmo-mir-285* | Domesticated | 17 | 0.01917 | 0.01354 | KC138075- KC138091 | This study |
|  | Wild | 11 | 0.03189 | 0.03538 | KC137982- KC137992 |  |
| *Bmo-mir-2794* | Domesticated | 17 | 0.01503 | 0.01221 | KC138109- KC138125 | This study |
|  | Wild | 11 | 0.01795 | 0.02818 | KC138004- KC138014 |  |
| *Bmo-mir-2795* | Domesticated | 17 | 0.02874 | 0.06584 | KC138126- KC138142 | This study |
|  | Wild | 10 | 0.02006 | 0.02216 | KC138015- KC138024 |  |
| *Bmo-mir-2822-1* | Domesticated | 17 | 0.01000 | 0.01286 | KC138143- KC138159 | This study |
|  | Wild | 11 | 0.01926 | 0.02294 | KC138025- KC138035 |  |
| *Bmo-mir-2823* | Domesticated | 17 | 0.03956 | 0.03373 | KC138160- KC138176 | This study |
|  | Wild | 11 | 0.03530 | 0.03794 | KC138036- KC138046 |  |
| *Bmo-mir-2827* | Domesticated | 16 | 0.00169 | 0.00197 | KC138177- KC138192 | This study |
|  | Wild | 9 | 0.00351 | 0.00401 | KC138047- KC138055 |  |
| *Bmo-mir-2831-2* | Domesticated | 16 | 0.01638 | 0.01435 | KC138193- KC138208 | This study |
|  | Wild | 10 | 0.02563 | 0.02618 | KC138056- KC138065 |  |
| *Bmo-mir-2837* | Domesticated | 17 | 0.02471 | 0.01869 | KC138209- KC138225 | This study |
|  | Wild | 9 | 0.03139 | 0.03146 | KC138066- KC138074 |  |

All we used genes are listed above. n: number of sample; π_silent_: π value for silent sites (synonymous and nocoding sites ); θ_silent_: θ value for silent sites (synonymous and nocoding sites).
