# Supplementary material for: Demographic history and gene flow during silkworm domestication
Source: BMC Evol Biol. 2014 Aug 14;14:185. doi: 10.1186/s12862-014-0185-0 (PMC4236568; doi:10.1186/s12862-014-0185-0)
Supplement: Additional file 9: Table S4. — Priors used for Approximate Bayesian Computation. [file s12862-014-0185-0-S9.doc]

**Table S4.** Priors used for Approximate Bayesian Computation

| Parameter | Range of prior | Distribution |
| --- | --- | --- |
| m | 0-10 | Uniform |
| θ_a_/θ_1_ | 0.5-2 | Uniform |
| θ_b2_/θ_1_ | 0-1 | Uniform |
| θ_b1_/θ_1_ | 0-1 | Uniform |
| θ_2_/θ_1_ | 0-1 | Uniform |
| τ_1_ | 0.0001-τ_D_ | Log Uniform |
| τ_2_ | 0.0001-τ_D_ | Log Uniform |
| τ_D_ | 0.0001-0.03 | Log Uniform |
