# Supplementary material for: Demographic history and gene flow during silkworm domestication
Source: BMC Evol Biol. 2014 Aug 14;14:185. doi: 10.1186/s12862-014-0185-0 (PMC4236568; doi:10.1186/s12862-014-0185-0)
Supplement: Additional file 10: Table S5. — Parameters of all hypothesized models. [file s12862-014-0185-0-S10.doc]

**Table S5.** Parameters of all hypothesized models

| Parameter | Parameter description |
| --- | --- |
| θ_a_ | The mutation rate of ancestral population |
| θ_1_ | The mutation rate of wild silkworm population (present-day) |
| θ_2_ | The mutation rate of domesticated silkworm population (present-day) |
| θ_b1_ | The mutation rate of wild silkworm population in historical period of time |
| θ_b2_ | The mutation rate of domesticated silkworm population during bottleneck |
| M_1_ | The mutation scaled effective migration rate mutation, from wild to domesticated silkworm |
| M_2_ | The mutation scaled effective migration rate mutation, from domesticated to wild silkworm |
| τ_D_ | The divergence time between the domesticated and wild silkworm population |
| τ_1_ | The assumed expansion time of wild silkworm population |
| τ_2_ | The end time of the domesticated silkworm during bottleneck |

Parameters of three hypothesized migration models are shown above. All θ parameters can be converted to *N*s (the effective population sizes, *N*_a_, *N*_2_, *N*_1_, *N* _b1_, *N* _b2_). The Conversion procedure is based on the equation θ = 4*N*μ . All τ parameters can also be converted to *T*s (the time in years, *T*_D_, *T*_1_, *T*_2_). Conversion procedure is based on the equation *T* = 4*N*τ. Gene flow rates M_1_ and M_2_ are scared by the neutral mutation rate μ (M = m/μ, m, the fraction of each subpopulation made up of new migrants each generation)
